# Factors influencing sedentary behaviours after stroke: findings from qualitative observations and interviews with stroke survivors and their caregivers

**DOI:** 10.1186/s12889-020-09113-6

**Published:** 2020-06-19

**Authors:** Jennifer Hall, Sarah Morton, Claire F. Fitzsimons, Jessica Faye Hall, Rekesh Corepal, Coralie English, Anne Forster, Rebecca Lawton, Anita Patel, Gillian Mead, David J. Clarke

**Affiliations:** 1 Academic Unit for Ageing and Stroke Research, University of Leeds, Bradford Royal Infirmary, Bradford, BD9 6RJ UK; 2 Bradford Institute for Health Research, Bradford Teaching Hospitals Foundation Trust, Bradford, UK; 3 Centre for Clinical Brain Sciences, University of Edinburgh, Edinburgh, UK; 4 Physical Activity for Health Research Centre, University of Edinburgh, Edinburgh, UK; 5 School of Health Sciences and Priority Research Centre for Stroke and Brain Injury, University of Newcastle, Newcastle, Australia; 6 School of Psychology, University of Leeds, Leeds, UK; 7 Anita Patel Health Economics Consulting Ltd, London, UK

**Keywords:** Stroke, Rehabilitation, Sedentary behaviour, Intervention development, Hospital, Community, Behaviour change, Sitting, Standing, Movement

## Abstract

**Background:**

Stroke survivors are more sedentary than healthy, age-matched controls, independent of functional capacity. Interventions are needed to encourage a reduction in overall sedentary time, and regular breaks in prolonged periods of sedentary behaviour. This study captured the views and experiences of stroke survivors and their caregivers related to sedentary behaviour after stroke, to inform the development of an intervention to reduce sedentary behaviour.

**Methods:**

Mixed-methods qualitative study. Non-participant observations were completed in two stroke services, inclusive of inpatient and community settings in the United Kingdom. Semi-structured interviews were conducted with stroke survivors and their caregivers (if available) at six- or nine-months post-stroke. Underpinned by the capability, opportunity and motivation (COM-B) model of behaviour change, observational data (132 h) were analysed thematically and interview data (*n* = 31 stroke survivors, *n* = 12 caregivers) were analysed using the Framework approach.

**Results:**

Observation participants differed in functional ability whereas stroke survivor interviewees were all ambulant. Six themes related to sedentary behaviour after stroke were generated: (1) sedentary behaviour levels and patterns after stroke; (2) the physical and social environment in the stroke service and in the home; (3) standing and movement capability after stroke; (4) emotion and motivation after stroke; (5) caregivers’ influence on, and role in influencing stroke survivors’ sedentary behaviour; and (6) intervening to reduce sedentary behaviour after stroke. Capability, opportunity and motivation were influenced by the impact of the stroke and caregivers’ inclination to support sedentary behaviour reduction. Stroke survivors reported being more sedentary than they were pre-stroke due to impaired balance and co-ordination, increased fatigue, and reduced confidence in mobilising. Caregivers inclination to support stroke survivors to reduce sedentary behaviour depended on factors including their willingness to withdraw from the caregiver role, and their perception of whether the stroke survivor would act on their encouragement.

**Conclusions:**

Many stroke survivors indicate being open to reducing sedentary behaviour, with appropriate support from stroke service staff and caregivers. The findings from this study have contributed to an intervention development process using the Behaviour Change Wheel (BCW) approach to develop strategies to reduce sedentary behaviour after stroke.

## Background

Greater time spent in sedentary behaviours (waking time spent sitting/lying/reclining with low energy expenditure (< 1.5 metabolic equivalents) [1]) is associated with higher cardiovascular disease incidence and mortality in the general population [2]. Negative associations have also been shown in relation to depression and anxiety [3, 4], physical function [5] and frailty [6]. Prevalence of sedentary behaviours after stroke is high in both inpatient and community settings (94% of the day spent sedentary on an acute stroke unit [7], 74% in a rehabilitation hospital [8] and 75% in community settings [9]). This volume of sedentary time places stroke survivors in the highest quartile of cardiovascular disease risk reported in epidemiological studies [5]. To counteract the detrimental effects of high levels of sedentary behaviours a relatively high level of moderate intensity physical activity (60–75 min/day) is recommended [10]. This level of activity is unlikely to be achieved in stroke survivors who have very low level of moderate/vigorous activity (4.9 min/day) [9] and there is evidence that step count might fall, rather than increase from the sub-acute to the chronic phase (5535 to 4078 steps/day) [11]. Stroke survivors tend to accumulate their sedentary time in longer uninterrupted periods of sedentary time in comparison to healthy age-matched controls [8, 11], which also leads to increased negative health risks [12].

Sedentary behaviours have been shown to be amenable to change in the general population, with a recent meta-analysis showing a reduction in daily sitting time of 30 min/day [13]. However, evidence from stroke survivors is limited and inconclusive with one Canadian home-based feasibility study (single group, *N* = 34) showing promising findings in relation to a reduction in sedentary time post intervention [14], and one community-based Australian phase II pilot randomised controlled trial (*N* = 35) demonstrating it is safe and feasible to intervene [15]. No interventions delivered in the inpatient stroke service setting have been published to date. Intervention development work with robust theoretical underpinning is needed to increase the likelihood of the intervention being efficacious [16, 17]. Qualitative work is required to develop a nuanced understanding of the barriers and facilitators to reducing sedentary behaviour in hospital and at home, to inform the development of appropriate interventions [18].

One previous qualitative study presented the perspectives of ambulatory stroke survivors on sedentary behaviour up to 12 years after stroke (*N* = 13) and showed limited awareness of health risks of sedentary behaviours with perceived barriers to behaviour change including motor impairments, fatigue, cognitive problems, and lack of motivation [19]. Caregivers play an important role in providing practical and emotional support to stroke survivors [20] and may influence stroke survivors’ ability and motivation to reduce sedentary behaviour. However, no interviews with caregivers of stroke survivors focused on sedentary behaviour have been conducted to date. Therefore, qualitative work with stroke survivors, who have experienced a stroke within the last 12 months, and their caregivers, is required. Behavioural theory is useful for examining the range of factors influencing specific health-related behaviours, to inform the development of appropriate interventions [21]. The COM-B model proposes that capability, opportunity, and motivation interact to generate behaviour [22]. Capability refers to an individual’s psychological and physical ability to engage in the behaviour, whereas opportunity relates to physical and social environmental factors. Motivation includes reflective decision-making processes, as well as automatic processes, based on emotional responses and associative learning and habit [22].

To inform the development of an intervention to reduce sedentary behaviour after stroke the aim of this study was firstly, to develop understanding of contemporary practice in terms of post-stroke sedentary behaviours (sitting, lying, reclining), standing behaviours, and physical activity behaviours, in two inpatient stroke units and in linked community services. Secondly, we sought to capture the views and experiences of a range of stroke survivors on sedentary behaviour, along with the views and perspectives of their caregivers. We also interviewed staff members from inpatient and community services; these findings are reported separately. Guided by the COM-B model of behaviour change [22], this study aimed to explore capability, opportunity and motivation to in relation to sedentary behaviours after stroke.

## Methods

The study was conducted in two inpatient stroke units (one mixed acute-rehabilitation unit, one rehabilitation unit) with linked community stroke services. Site one was in the North of England and site two in the South of Scotland. The study received a favourable ethical opinion from the Health Research Authority, Yorkshire & the Humber - Bradford Leeds Research Ethics Committee [17/YH/0236] and the Scotland A Research Ethics Committee [17/SS/0099].

We implemented a mixed-methods approach combining non-participant observations and semi-structured interviews. The approach was both sequential (observations occurring prior to interviews and shaping interview questions) and convergent, with equal weighting placed on the two methods, with the purpose of attaining a more complete understanding [23]. Non-participant observations, using an established qualitative framework [24]; see supplementary file 1, were conducted in inpatient and community settings. In the inpatient settings, observation sessions were conducted on weekday and weekend mornings, afternoons and evenings, and lasted between 4 and 5 h on each occasion for the equivalent of 2 weeks in each location. Observations were conducted in different public areas of stroke units, in group, or individual therapy sessions, in day hospital community stroke services and in stroke survivors’ own homes. We focused initially on stroke unit contexts, including the built environment and facilities and how and where patients’ time was spent. We sought to understand staff practices and daily routines as these were experienced by stroke survivors and caregivers. Alongside this, focused observations with individual stroke survivors permitted the collection of more detailed information about patient specific activity, for example with therapists or nurses. In community settings, we conducted focused observations with individual stroke survivors and their caregivers, observing interactions on multiple occasions with therapists or nurses during home visits, or interactions with therapists and/or other health and social care staff during stroke survivor and caregiver visits to community stroke services. The observation sessions varied in length depending on the type of interaction observed.

During all observations, researchers did not take part in activities or in therapy, medical or nursing interactions. Field notes were recorded contemporaneously; the observation framework was used as a sensitising framework to guide, but not constrain, note taking. Activities observed included support for personal care, transfers, balance and mobility, walking practice, upper limb activities. The nature and focus of interactions between stroke survivors, staff and caregivers were summarised in the field notes. For general observations in public areas in stroke units and day hospital settings the site Principal Investigator and all stroke service staff provided written consent. In addition, a process consent approach was adopted to ensure any persons present in an area being observed were provided with information on the purpose of the observations and had an opportunity to request that observations did not take place in a given area at a given time. Written informed consent was obtained from all staff, stroke survivors and carers for direct observations of individual or group therapy sessions.

Audio-recorded semi-structured interviews were conducted with stroke survivors and their caregivers at either six- or nine-months post-stroke, as at these time points neurological recovery would be well advanced and adjustment to post-stroke life stabilising. Interview topic guides were developed and structured to explore capability, opportunity and motivation [1] in relation to sedentary behaviours after stroke (see Supplementary file 2). The theoretical domains framework was utilised to inform development of the interview guide [25]. We also asked stroke survivors and caregivers for their views on realistic and practical ways that time spent sitting could be reduced and time spent upright or moving could be increased, to inform intervention development. Stroke survivor and caregiver views on participation in a programme related to sedentary behaviour reduction were explored along with views on the timing of such a programme, i.e. in the inpatient and community setting or only after discharge from hospital. Interviews took place in participants’ own homes and involved stroke survivors and caregivers in a single interview. Written consent was obtained from all participating stroke survivors and caregivers.

As far as was possible participants were selected purposively to attain a sample that is heterogeneous with respect to characteristics such as gender, age, and stroke severity. Inclusion criteria included being aged 16 years or over, English-speaking, and able and willing to provide informed consent or, for observations only, for whom a consultee declaration was provided. Participants in the observational element of the study were also required to have a confirmed primary diagnosis of new stroke and be currently receiving care or treatment in a participating stroke service. Interview participants were required to be community-dwelling, have received a confirmed diagnosis of new stroke in the last 5–10 months, and currently, or have previously received post-stroke care from a participating stroke service. All stroke survivors completed questionnaires at the time of recruitment including the Functional Ambulatory Categories (FAC; a six-point scale (1–6) with a higher score indicating better mobility) [26], the Nottingham Extended Activities of Daily Living (NEADL; scores range from 22 to 66 with a higher score indicating more independence in activities of daily living) [27], Barthel index (observation participants only; scores range from 0 to 20 with a higher score indicating higher functional ability) [28] and a Sedentary Behaviour Visual Analogue Scale (SB-VAS; 0–10 scale with a higher number indicating increased time spent sedentary, interview participants only) for the purpose of characterising the sample.

Observational data were analysed thematically. One researcher from each site completed thematic analysis of all observational data for that site. This was then reviewed by a senior researcher at each site, and summary memos outlining routinely observed stroke survivor, carer and staff behaviours related to sitting and upright time and factors influencing capability, opportunity and motivation to change sitting and upright time were developed. Interviews were transcribed verbatim and managed alongside field-notes from observations in QSR-NVivo11 (QSR International Pty Ltd., 2016). Interview data were analysed using the Framework approach [29]; Table 1. Six researchers (JH, SM, JFH, RC, DJC, CF) were involved in a progressive, inductive open coding process; two transcripts were coded by all six researchers, four by three researchers, and six by two researchers (step 1). A coding framework was developed and agreed upon (step 2), and then utilised to code all interviews (step 3). This coding framework included nine main themes, with 40 sub-themes. One researcher coded all interviews from a site (Yorkshire JH, Edinburgh SM); five of the interviews were independently double coded (JFH). Researchers met to review coding consistency before creating Framework matrices (step 4). Working in pairs, the same six researchers created summary memos (three per pair) based on interpretation of the descriptive thematic data charted in the Framework matrices. The components of the COM-B model [1; see supplementary file 3] were utilised to interpret the thematic data and develop the summary memos. Specifically, the researchers focused on considering how capability, opportunity and motivation factors influence sedentary behaviour, when interpreting the data. The summary memos from the interview and observational data analysis were compared and reviewed by all researchers to agree the analytical themes reported below (step 5). A convergent mixed-methods approach was taken, meaning that equal weighting was placed on the data and findings from the observational and interview methods within this final stage of analysis, to attain a more complete understanding than could be gained from using either method in isolation [23]. However, some themes from the observational findings including the organisation of the stroke service and work, and routine staff practices, approaches and behaviours, were not included within the final step of the framework analysis. This is because these findings are presented within a separate paper focused on understanding the role of stroke service staff in supporting patients to reduce sedentary behaviour (in preparation). This final analytical step was deductive in nature, guided by the COM-B model. The researchers thematically re-organised data from across the summary memos according to stroke survivors and caregivers capability, opportunity and motivation to (support stroke survivors to) reduce sedentary behaviour.

**Table 1:** Stages in Framework Analysis [26]

1) Familiarisation with the data *(reading, and re-reading field-notes, transcripts, memos)*	
2) Identifying a thematic framework *(researchers jointly developing a set of codes organised into categories to manage and organise the data)*	
3) Indexing *(systematically applying the thematic framework to the whole data set)*	
4) Charting *(entering data into Framework matrices: spreadsheets containing cells into which summarised data are entered by codes (columns) and cases (rows))*	
5) Mapping and interpretation *(interpretive concepts or propositions describing or explaining aspects of the data are the final output of the analysis)*	

## Results

Forty-eight stroke survivors and sixteen caregivers participated in the study. One hundred and thirty-three hours of observations were completed involving focused observations of 19 stroke survivors and five caregivers across both settings and locations. Observations took place between October 2017 and March 2018. Interviews ranged from 23 min to 173 min and lasted an average of 66 min. Stroke survivor interviewees, 31 in total, were approximately 6-months post-stroke (*n* = 20, 8 female, mean 75 years old, FAC = 5.6, NEADL = 49, SB VAS = 6) or 9-months post-stroke (*n* = 11, 5 female, mean 73 years old, FAC = 5.6, NEADL = 47, SB VAS = 7). All interview participants were ambulant whereas observation participants varied in mobility status (FAC scores range from 1 to 6; Barthel scores range from 7 to 20). See Table 2 for an overview of stroke survivor and caregiver observation individual participant characteristics, and Table 3 for an overview of stroke survivor and caregiver interviewee participant characteristics.

**Table 2:** Stroke survivor and caregiver observation participant characteristics table

Site	Stroke survivor or caregiver	Pseudonym	Related stroke survivor	Relationship to stroke survivor	Observation setting	Gender	Age	Occupational status	Barthel score	FAC
Yorkshire	Stroke survivor	Olivia	–	–	Inpatient	F	70–79	Retired	19	6
Yorkshire	Stroke survivor	Florence	–	–	Inpatient	F	70–79	Retired	7	1
Yorkshire	Stroke survivor	Charlotte	–	–	Community	F	70–79	Retired	19	5
Yorkshire	Stroke survivor	George	–	–	Community	M	80–89	Retired	7	1
Yorkshire	Stroke survivor	Jack	–	–	Community	M	30–39	Employed	12	4
Yorkshire	Stroke survivor	Oliver	–	–	Inpatient & community	M	60–69	Employed	15	4
Yorkshire	Stroke survivor	Harry	–	–	Inpatient & community	M	80–89	Retired	16	3
Yorkshire	Caregiver	Joseph	Olivia	Spouse	Inpatient	M	80–89	Retired	–	–
Yorkshire	Caregiver	David	Florence	Son	Inpatient	M	50–59	Employed	–	–
Yorkshire	Caregiver	Eliza	George	Daughter	Community	F	60–69	Retired	–	–
Yorkshire	Caregiver	Matilda	Jack	Co-habiting partner	Community	F	40–49	Employed	–	–
Yorkshire	Caregiver	Joshua	Harry	Son	Inpatient & Community	M	40–49	Employed	–	–
Edinburgh	Stroke survivor	Jaxon	–	–	Inpatient	M	80–89	Retired	7	3
Edinburgh	Stroke survivor	Luca	–	–	Inpatient	M	40–49	Employed	20	6
Edinburgh	Stroke survivor	Matthew	–	–	Inpatient	M	60–69	Employed	7	1
Edinburgh	Stroke survivor	Emma	–	–	Community	F	40–49	Employed	20	6
Edinburgh	Stroke survivor	Harvey	–	–	Community	M	60–69	Retired	20	6
Edinburgh	Stroke survivor	Amber	–	–	Community	F	40–49	Employed	18	4
Edinburgh	Stroke survivor	Harley	–	–	Community	M	50–59	Employed	17	3
Edinburgh	Stroke survivor	Reggie	–	–	Community	M	70–79	Retired	16	2
Edinburgh	Stroke survivor	Tommy	–	–	Community	M	50–59	Retired	15	5
Edinburgh	Stroke survivor	Mollie	–	–	Community	F	50–59	Employed	16	2
Edinburgh	Stroke survivor	Maisie	–	–	Inpatient & Community	F	60–69	Retired	20	6
Edinburgh	Caregiver	Holly	Matthew	Partner	Inpatient	F	–	–	–	–

**Table 3:** Stroke survivor and caregiver interviewee participant characteristics table

Site	Pseudonym	Stroke survivor or caregiver	Related stroke survivor	Relationship to stroke survivor	Interview time point	Gender	Age	Occupational status	FAC	NEADL	SBVAS
Yorkshire	Oscar	Stroke survivor	–	–	6 months	M	80–89	Retired	6	54	–
Yorkshire	Imogen	Stroke survivor	–	–	6 months	F	70–79	Retired	5	34	6
Yorkshire	Noah	Stroke survivor	–	–	6 months	M	90–99	Retired	6	52	9
Yorkshire	Henry	Stroke survivor	–	–	6 months	M	80–89	Retired	5	56	6
Yorkshire	Harry	Stroke survivor	–	–	6 months	M	80–89	Retired	6	63	2
Yorkshire	Olivia	Stroke survivor	–	–	6 months	F	70–79	Retired	6	43	6.5
Yorkshire	Alexander	Stroke survivor	–	–	6 months	M	80–89	Retired	5	45	9
Yorkshire	Edward	Stroke survivor	–	–	6 months	M	80–89	Retired	6	63	4
Yorkshire	Arthur	Stroke survivor	–	–	6 months	M	70–79	Retired	6	53	6
Yorkshire	Rosie	Stroke survivor	–	–	6 months	F	50–59	Unemployed	4	16	7.5
Yorkshire	Thomas	Stroke survivor	–	–	9 months	M	60–69	Retired	4	14	9
Yorkshire	Charlie	Stroke survivor	–	–	9 months	M	50–59	Unemployed	5	35	–
Yorkshire	Oliver	Stroke survivor	–	–	9 months	M	60–69	Employed	6	57	5
Yorkshire	William	Stroke survivor	–	–	9 months	M	80–89	Retired	6	–	–
Yorkshire	Evelyn	Stroke survivor	–	–	9 months	F	80–89	Retired	6	41	8
Yorkshire	Charlotte	Stroke survivor	–	–	9 months	F	70–79	Retired	6	40	8
Yorkshire	Joseph	Caregiver	Olivia	Spouse	6 months	M	80–89	Retired	–	–	–
Yorkshire	Joshua	Caregiver	Harry	Son	6 months	M	40–49	Employed	–	–	–
Yorkshire	Freya	Caregiver	Thomas	Spouse	9 months	F	60–69	Retired	–	–	–
Yorkshire	Isabelle	Caregiver	Charlie	Partner	9 months	F	50–59	Employed	–	–	–
Yorkshire	James	Caregiver	Imogen	Spouse	6 months	M	70–79	Retired	–	–	–
Yorkshire	Scarlett	Caregiver	William	Daughter	9 months	F	50–59	Retired	–	–	–
Yorkshire	Elizabeth	Caregiver	Henry	Spouse	6 months	F	70–79	Retired	–	–	–
Yorkshire	Daisy	Caregiver	Arthur	Spouse	6 months	F	70–79	Retired	–	–	–
Yorkshire	Samuel	Caregiver	Rosie	Partner	6 months	M	50–59	Employed	–	–	–
Edinburgh	Luke	Stroke survivor	–	–	6 months	M	80–89	Retired	6	66	4.5
Edinburgh	Zara	Stroke survivor	–	–	6 months	F	–	Retired	6	–	10
Edinburgh	Jude	Stroke survivor	–	–	6 months	M	60–69	Retired	6	62	10
Edinburgh	Harvey	Stroke survivor	–	–	6 months	M	80–89	Retired	6	66	6
Edinburgh	Frankie	Stroke survivor	–	–	6 months	M	90–99	Retired	4	31	8
Edinburgh	Heidi	Stroke survivor	–	–	6 months	F	80–89	Retired	6	57	5.5
Edinburgh	Gracie	Stroke survivor	–	–	6 months	F	70–79	Retired	6	53	7.3
Edinburgh	Luna	Stroke survivor	–	–	6 months	F	80–89	Retired	6	32	7.4
Edinburgh	Albert	Stroke survivor	–	–	6 months	M	40–49	Employed	6	28	5.5
Edinburgh	Emma	Stroke survivor	–	–	6 months	F	40–49	Employed	6	63	4
Edinburgh	Aria	Stroke survivor	–	–	9 months	F	80–89	Retired	5	53	3
Edinburgh	Maria	Stroke survivor	–	–	9 months	F	50–59	Retired	6	63	7.5
Edinburgh	Jayden	Stroke survivor	–	–	9 months	M	70–79	Retired	6	54	7
Edinburgh	Anna	Stroke survivor	–	–	9 months	F	70–79	Employed	–	60	4.5
Edinburgh	Jenson	Stroke survivor	–	–	9 months	M	70–79	Retired	6	66	7
Edinburgh	Nancy	Caregiver	Jayden	Spouse	9 months	F	–	Retired	–	–	–
Edinburgh	Martha	Caregiver	Frankie	Spouse	6 months	F	–	Retired	–	–	–
Edinburgh	Stanley	Caregiver	Heidi	Spouse	6 months	M	–	Retired	–	–	–

The analysis produced six themes that contribute to an understanding of sedentary behaviours in stroke survivors. The themes are: (1) sedentary behaviour levels and patterns after stroke, (2) the physical and social environment in the stroke service and in the home, (3) standing and movement capability after stroke, (4) emotion and motivation after stroke, (5) caregivers’ influence on, and role in influencing, stroke survivors’ sedentary behaviour, and (6) intervening to reduce sedentary behaviour after stroke. An illustration of how the themes map to the COM-B model is provided in Supplementary File 3. In the anonymised direct quotations which follow, all participants are referred to by pseudonyms.

### Sedentary behaviour levels and patterns after stroke

All stroke survivors indicated that they spent more time sitting and lying down in the months following their stroke than prior to the event. This theme describes levels and patterns of behaviour in the inpatient setting and at home.

#### Behaviour in the inpatient setting

Observations conducted in both stroke units indicated that inpatient stroke survivors spend most of the day in sedentary behaviours, either lying in bed or sitting in a bedside chair. These extended periods of sedentary behaviour were only interrupted when stroke survivors attended therapy, during washing and dressing or when being taken to or from the toilet or bathrooms by staff, or if taken off the unit to a café or garden by family members. Stroke survivors were most commonly observed sitting or lying down while engaging in no other (cognitive, social etc.) activity. During informal interactions with the researchers, some stroke survivors suggested that they felt this prolonged sitting and lying had a restorative function related to fatigue, whereas others perceived that it lacked purpose and found it boring.

Other sedentary activities inpatient stroke survivors were observed engaging in included reading, using electronic devices, and talking with visitors. Observations indicated that stroke survivors broke up prolonged sedentary behaviour less frequently during the evenings, compared to the morning and afternoon. The most frequently observed non-sedentary activity was sleeping.

#### Behaviour following return home from the inpatient setting

All interviewees voiced that they engaged in a wider range of activities involving standing and moving upon return home from the inpatient setting. This included self-care, household, and social (within and outside the home) activities:

*“Maybe after I go for a walk… having half-an-hour reading and [then] go upstairs and do my ironing you know… then I make a meal and settle down. But that’s if I’m here, we like to go out for afternoon tea and that sort of thing, to the garden centre”* (Aria, 9-months post-stroke, 84 years, FAC = 5)

Stroke survivors discussed relatively defined pre-stroke patterns of activity and sedentary behaviour across the course of the day that tended to endure once returning home post-stroke. The most typical daily pattern articulated by stroke survivors was sedentary behaviour being broken up less frequently as the day progressed, with evenings often consisting of uninterrupted sitting activities. However, interviewees indicated that they were more sedentary than they were pre-stroke, and *some* were unable to engage in activities they enjoyed pre-stroke, such as driving, holidays, gardening and housework.

### The physical and social environment in the stroke service and in the home

The physical environment contributed to high levels of sedentary behaviour in the inpatient setting. On the mixed acute-rehabilitation stroke unit, many patients were observed to be connected to medical equipment that prevented them from standing and moving. In both services, small spaces, further hindered by the presence/storage of equipment in patient bays and corridors, made it more difficult for stroke survivors to move around the stroke unit.

Stroke survivor interviewees indicated that they were reluctant to ask hospital staff to supervise or assist activities involving standing and movement because they perceived staff to be under time pressures. When asked why he thought he spent so much time sitting down when he was in hospital, Oliver responded that: *“There weren’t enough staff around and I was immobile anyway with my leg”* (Oliver, 9-months post-stroke, 63 years, FAC = 6).

Some stroke survivor and caregiver interviewees believed that stroke survivors were more receptive to advice about movement provided by stroke service staff than by family and friends:

*“People do listen to the medical people… I think that incentivises people...the voice of authority”* (Maria, 9-months post-stroke, 59 years, FAC = 6)

The observational data showed that stroke survivors’ differing home environments contributed to individual differences in sedentary behaviour after stroke. The presence of environmental modifications, such as safety handles and stair lifts provided opportunities to move independently within and outside the home. Stroke survivors who lived in accommodation that they did not own were less able to make environmental adaptions, and appropriate organisational input was often required to gain permissions to do so.

### Standing and movement capability after stroke

Observational data revealed wide variation in capability to stand and move between stroke survivors depending on the severity and presentation of post-stroke impairment. Support required included physical assistance or supervision, or the use of walking aids, wheelchairs or hoists. Interviewees described physical impairments that continued to impact on their lives:

*”[I’m] not quite as able since I’ve had the stroke but things are getting better all the time, sort of like a week by a week I can notice that yes I can do something better than I could do before”* (Alexander, 6-months post-stroke, 82 years, FAC = 5)

At six- and nine-months post-stroke the impairments most commonly linked to reduced ability to stand and ambulate were impaired balance and co-ordination, coupled with (hemi)paresis:

*“I just feel a bit unsteady if I turn quickly, and that’s probably because my left leg isn’t as strong as it used to be”* (Jenson, 9-months post-stroke, 71 years, FAC = 6)

Post-stroke fatigue was also cited as a barrier to reducing and breaking up sedentary behaviour for some stroke survivors. Some stroke survivors reported feeling frustrated that fatigue was ‘forcing’ them to sit for longer than they desired:

*“If you get fatigue after a stroke you cannot over[come it]… like I love gardening, I still do, I can’t do it, I try but after sort of half-an-hour, three-quarters I’m totalled”* (Imogen, 6-months post-stroke, 72 years, FAC = 5)

*“It’s frustrating for me not to be able to get off my backside and do something”* (Jayden, 9-months post-stroke, 74 years, FAC = 6)

Other interviewees indicated balancing activities involving standing and movement with sitting to manage fatigue, and did not experience guilt when resting following engagement in an activity:

*“So, she said ‘once you get up and wash and everything, half an hour rest. If then you make the beds, half an hour rest’ … so actually it’s quite nice for me, it’s a bit of a new thing… where I can sit and I think, I don’t feel guilty”* (Olivia, 6-months post-stroke, 71 years, FAC = 6)

Upper-limb impairments limited opportunities for reducing sedentary behaviour, as stroke survivors reported being less able to engage in functional activities:

*“I can use me hand as a hand but I can’t use the fingers. I did the cooking, the cleaning, the washing, the ironing [before the stroke], I can’t do it now”* (Imogen, 6-months post-stroke, 72 years, FAC = 5)

The stroke event and associated impairments also reduced stroke survivors’ general confidence and perception of their capability to stand and move, particularly in relation to fear of falling, which was often linked to the environment (e.g. uneven ground, adverse weather conditions):

*“It were [sic] windy and I felt as though I could get blown over here. Well that would never have bothered me in the past”* (Thomas, 9-months post-stroke, 60 years, FAC = 4)

Most interviewees indicated that pre-existing health issues such as arthritis, made it difficult for them to stand and move both prior to, and following, their stroke:

*“I mean the hip tends to make walking a) uncertain and b) slightly painful…But that’s been, that’s not since the stroke, that’s been for two or three years now”* (Henry, 6-months post-stroke, 81 years, FAC = 5)

### Emotions and motivation after stroke

Some stroke survivors expressed that negative emotions linked to stroke-related impairments and the perceptions of others prevented them from leaving the home and engaging in previously valued activities:

*“I hated every minute of it, ‘Please take me home’. But that was because I was thinking [of] how I looked to other people”* (Charlotte, 9-months post-stroke, 77 years, FAC = 6)

Stroke survivors varied in their motivation to engage in rehabilitation activities. Some expressed a desire to return to valued and independent activities they engaged in pre-stroke:

*“I still do those [therapy exercises] twice a day. I just want to get back 100%, if I can, you know? As I say, my main ambition is in August [to] cut my own hedges”* (Harry, 6-months post-stroke, 81 years, FAC = 6)

Other stroke survivors were less motivated to engage in rehabilitation activities, saying things like they: *“can’t be bothered”* (Zara, 6-months post-stroke).

Stroke survivors indicated that they valued activities including watching TV, reading, crafts, and other cognitive activities, which usually occur in a seated posture. Interviewees were more concerned with whether they were *occupied* than if they were being sedentary or active, and interviewees’ narratives indicated that sedentary behaviours involving cognitive engagement, such as reading, were valued over ‘passive’ sedentary behaviours:

*“I think maybe half of the sedentary things is good providing you’re keeping your brain going and you’re not sitting there just thinking of nothing”* (Maria, 9-months post-stroke, 59 years, FAC = 6)

Some interviewees said the stroke motivated them to make changes to their health behaviour, usually to minimise the risk of stroke recurrence:

*“I wasn’t one for going to the gym, smoked 40 a day, haven’t smoked since the day I had the stroke, and that’s it”* (Albert, 6-months post-stroke, 42 years, FAC = 6)

Other interviewees indicated that the stroke did not prompt them to consider lifestyle changes. Reasons cited included disillusionment with exhibiting a ‘healthy lifestyle’, especially in stroke survivors who perceived they had a ‘healthy lifestyle’ pre-stroke, and a perception that changes were not required as they had already modified their lifestyle to manage other conditions:

*“That’s the thing that’s bugging her really as well, that all her life… she’s eaten well, she doesn’t drink, she doesn’t smoke and then she had a stroke”* (James, caregiver, 74 years).

*“I’m careful with the sugar because me [sic] sister… she went blind with diabetes… if you don’t watch it, yeah, you’re going to suffer the effects, aren’t you?”* (Edward, 6-months post-stroke, 80 years, FAC = 6)

### Caregivers’ influence on, and role in influencing, stroke survivors’ sedentary behaviour

The observational and interview data highlighted current caregiver behaviour that influences stroke survivors’ sedentary behaviour, and barriers and facilitators related to supporting the stroke survivor to reduce sedentary behaviour, connected to the caregiver role and circumstances, and the perceived influence on the stroke survivor and their relationship with the stroke survivor.

#### Current caregiver support behaviour

Caregiver interviewees provided examples of things they currently do to facilitate stroke survivors spending less time sitting. For example, some caregivers said they encouraged, supervised, or physically supported the stroke survivor to engage in activities involving standing and movement, including personal care activities and household tasks:

*“It’s important I’m handy while she’s dealing with the kettle and while she’s pouring the water in the cups and that*” (Joseph, caregiver, 83 years)

Caregivers also reported engaging in joint activities involving standing and moving with the stroke survivor, for example, walking, attending social groups, and visiting friends:

*“He cut back on his workload which I'm glad, so I'm able to go out and about”* (Heidi, 6-months post-stroke, 82 years) … “*We've done more of it since she had the stroke*” (Stanley, caregiver)

#### The caregiver ‘role’, responsibilities and circumstances

Some caregiver interviewees spoke of an increased sense of responsibility for the stroke survivor following their stroke, linked to their post-stroke impairments, which generated a lot of additional ‘work’, including supervising or helping with daily tasks. Whilst some caregivers said they had enough time to dedicate to supporting the stroke survivor, others had to balance their caregiver role with various other responsibilities including employment and other caring roles:

“*But then some people, they can’t do it because they’re too busy, they’ve got, you know, other things to do… most of the day I’m not here because I’m at work, you see*” (Isabelle, caregiver, 52 years)

Some caregivers spoke of a natural process of withdrawing from ‘caring’ activities to facilitate independence in the stroke survivor, alongside improvements in the stroke survivor’s capabilities:

“*There are now quite a few times when I sort of say “tell me when you want help”, so I’ll let him persevere a little bit more”* (Freya, caregiver, 60 years)

However, some caregivers expressed a feeling of apprehension related to withdrawing from the caring role, for example, Freya said: “*letting go is quite hard because I want him to be safe”* (Caregiver, 60 years). A reluctance to, or uncertainty about when to, withdraw from the caregiver role could limit stroke survivors’ opportunities, and capability, to reduce sedentary behaviour.

#### Perceived consequences of supporting sedentary behaviour reduction

Some caregivers of stroke survivors who reported significant physical post-stroke impairments were reluctant to encourage the stroke survivor to reduce sedentary behaviour as they thought additional standing and movement may cause the stroke survivor unnecessary pain or discomfort:

*“Until her leg and her arm and her balance improve… to be honest it would be a waste of time because it would be inflicting pain on her, as things stand”* (James, caregiver, 74 years)

Caregivers of stroke survivors who were not capable of, or motivated to, reduce sedentary behaviour were disinclined to encourage the stroke survivor to reduce sedentary behaviour, as they felt that the stroke survivor would not act on the encouragement:

*“I tell him, look, move around, do the squats at the kettle… I don't know how many times I've told him that, but it doesn't seem to register, or he doesn't want it to register… it’s about how receptive someone is going to be to advice”* (Scarlett, caregiver, 53 years)

Caregivers did not want to negatively affect their relationship with the stroke survivor, for example, by being perceived as ‘nagging’:

*“I just think ‘oh I don’t want to be this nagging sort of person’… I don’t want the time that I’ve got with Thomas to be spent trying to make him do things he doesn’t want to do, I want the time just to be happy together*” (Freya, caregiver, 60 years)

### Intervening to reduce sedentary behaviour after stroke

The theme below describes whether and why stroke survivors would participate in an intervention, and their preferences related to timing, content and delivery of an intervention to reduce sedentary behaviour after stroke.

#### Views on taking part in a post-stroke intervention to reduce sedentary behaviour

Approximately half of the stroke survivors interviewed expressed an interest in taking part in an intervention. Reasons for wanting or not wanting to participate align with findings contained in the earlier themes, such as a perceived incapability to stand more, and a perception that standing more may facilitate recovery.

Receptiveness to participation also varied depending on stroke survivors’ awareness of and beliefs about the relationship between sedentary behaviour and health. A minority of interviewees mentioned physical health factors, including *“[increased] blood pressure”* (Scarlett, caregiver, 53 years), and *“[reduced] circulation”* (Charlie, 9-months post-stroke, 55 years, FAC = 5) when asked about how they thought too much sitting might affect their health. Some interviewees said that sitting - without being otherwise engaged - sometimes led them to ruminate about the impact of the stroke, which had a knock-on, negative impact on their mood:

*“You can become depressed by just sitting, if you’re on your own… go and do something, you know”* (Edward, 6-months post-stroke, 80 years, FAC = 6)

Some interviewees said they were unaware of how sitting affected health and other (typically older) interviewees recognised - but did not value - a relationship between sitting and health:

*“I wouldn't anticipate the [health] benefits [of reducing sedentary behaviour] at all… absolute poo… I just continue as I feel”* (William, 9-months post-stroke, 88 years, FAC = 6)

Stroke survivors who were aware of physical and / or mental health risks of sedentary behaviour, and who placed value on adjusting their behaviour to improve their health, were more likely to voice a desire to reduce sedentary behaviour after stroke.

#### When to intervene to reduce sedentary behaviour after stroke: timing considerations

Interviewees reported mixed views relating to timing preferences for a post-stroke sedentary behaviour reduction intervention. Approximately half of the interviewees thought that an intervention should commence almost immediately post-stroke, in the hospital setting, for reasons including filling the time and a perception that it would use useful to frame the intervention as contributing to stroke rehabilitation:

*“You’ve nothing else to do in hospital”* (Aria, 9-months post-stroke, 84 years, FAC = 5)

*“It’s as well to introduce it… at the time that they are recovering from the stroke, because that actually helps you to sell it”* (Henry, 6-months post-stroke, 81 years, FAC = 5)

However, other interviewees indicated that the immediate post-stroke period can be *“overwhelming”* (Frankie, 6-months post-stroke, 92 years, FAC = 4), and that stroke survivors might be able to engage with an intervention once they have returned home and have *“come to terms with it [the stroke]”* (Maria, 9-months post-stroke, 59 years, FAC = 6) and are physically able to stand.

#### How to support sedentary behaviour reduction: content and delivery preferences

When asked about preferences for a sedentary behaviour reduction intervention, a common suggestion was a group exercise class, partly due to the social nature of such activities:

*“The contact that you make with other people is one of the things that actually persuades you to be more actively involved I think… ‘If it means I’ll meet you again mate, I’ll come’, sort of thing”* (Henry, 6-months post-stroke, 81 years, FAC = 5)

Interviewees also felt that information provision around the health consequences of sedentary behaviour, and of ways to integrate more standing and movement into the day, was required:

*“Information, because that’s encouragement, encouraging not to rest for too long. Maybe some suggestions of useful exercises”* (Gracie, 6-months post-stroke, 77 years, FAC = 6)

Interviewees felt that suggestions of purposeful activities, for example *“make[ing] a cup of tea”* (Aria, 9-months post-stroke, 84 years, FAC = 5) or *“discover[ing] some sort of craft… something you enjoy doing”* (Anna, 9-months post-stroke, 79 years) would be most acceptable to them as they were reluctant to engage in activities that were not meaningful.

Stroke survivors indicated that utilising reminders to stand, e.g. *“an alarm of some sort that makes you think, ‘oh I’ve been sitting too long’”* (Maria, 9-months post-stroke, 59 years, FAC = 6) would be useful for prompting standing, particularly for those with post-stroke memory impairments.

When caregivers were asked about how they could encourage or support the stroke survivor to sit less, one caregiver suggested prompting the stroke survivor to stand at regular intervals:

*“If you sent a text every half hour, ‘what you doing, are you up’, you know, stuff like that”* (Isabelle, caregiver, 52 years)

However, most caregiver interviewees were unable to identify ways they could support the stroke survivor to stand and move more:

*“I don’t know really [pause]. I can’t really think of anything”* (Elizabeth, caregiver, 75 years)

There was a recognition that an intervention would need to account for differences in stroke survivors’ capability and preferences, as *“everybody’s different… there’s no two stroke people the same”* (Albert, 6-months post-stroke, 42 years, FAC = 6).

## Discussion

This is the first paper to qualitatively explore stroke survivors’ sedentary behaviour using observation in stroke units and linked community services and interviews with stroke survivors and their informal caregivers. We identified a range of stroke-related and non-stroke-related factors influencing stroke survivors’ capability, opportunity, and motivation to reduce or break up sedentary behaviour; see supplementary file 3 for an illustration of how the findings align with the COM-B model. Staff attitudes, interactions and behaviour that implicate stroke survivors’ sedentary behaviour are explored in more detail in another paper (in preparation).

### Levels and patterns of sedentary behaviour after stroke

The stroke survivors in this study are highly sedentary in both hospital and home settings. These findings are consistent with evidence from an observational study that reported an average of 86.9% of waking hours in sedentary behaviour in the inpatient stroke service setting, based on heart rate and accelerometer data [30]. Numerous behavioural mapping studies have reported that patients engage in little physical activity on stroke units (e.g. [31, 32]). In the present study, stroke survivors reported an increase in standing and moving once they returned home. This is consistent with a recent observational study that found that stroke survivors spent an average of 45 fewer minutes sitting a day during the week they returned home, in comparison to their last week in hospital [33]. The present study indicates that stroke survivors engage in a wider range of self-care, household, and social activities once they are discharged from hospital. However, a recent review of sedentary behaviour and physical activity levels after stroke, including 103 studies, found that physical activity levels increase over time after a stroke, whereas time spent sedentary remains high [9].

Stroke survivors’ reported routines tended to involve more standing and moving in the earlier parts of the day, and more (uninterrupted) sedentary behaviour during the afternoon and evening, which is consistent with the emerging literature investigating temporal sedentary behaviour patterns after stroke [30, 34]. The findings highlight that stroke survivor’s value rest as part of the recovery process, particularly when they experience post-stroke fatigue, which may explain the high levels of sedentary behaviour in the evening. It is also important to recognise that engaging in *some* sedentary behaviour is normal and important during stroke recovery [19]. A recent feasibility study of a home-based intervention to reduce sedentary behaviour after stroke found that whilst sedentary behaviour was reduced following the intervention, this was largely replaced by sleep [14]. One of the aims of the intervention was to complete > 6000 steps per day, which could be too intense for many stroke survivors, and induce tiredness. Interventions should emphasise the importance of breaking up sedentary behaviour throughout the day with short bouts of light-intensity activity. Such pacing of activity may reduce the likelihood of tiredness causing stroke survivors to engage in uninterrupted sedentary behaviour for prolonged periods or replacing sedentary behaviour with sleep.

### Why are people sedentary after stroke?

The present study identified a range of factors influencing stroke survivors’ sedentary behaviour. Consequences of – and factors linked to - the stroke, including lower and upper limb impairments, perceptions related to recovery prospects, and confidence, all influenced stroke survivors’ capability and propensity to reduce sedentary behaviour, which helps explain the high levels of sedentary behaviour in this patient group. Negative beliefs about standing and moving capability, regardless of actual capability, were identified as a barrier to spending less time sedentary. These factors are consistent with findings from interviews exploring the perceptions of ambulatory stroke survivors who had their stroke between 3 months and 12 years prior to the interview [19]. In a cross-sectional study, it was reported that having moderate stroke symptoms was associated with 1.4 h more sedentary behaviour/day, compared with stroke survivors who had no symptoms [33]. Whilst it might be expected that lower-limb impairment is most closely associated with levels of sedentary behaviour, a cross-sectional study found that more severe upper-limb impairment is associated with higher levels of sedentary behaviour after stroke [35]. Data in the present study suggests that stroke survivors have limited opportunity to engage in activities involving standing and moving when their upper limb is impaired, as they are less able to engage in functional activities, such as cooking. An individual’s physical and emotional capabilities and opportunities for engaging with standing and movement activities related to the stroke event requires consideration when tailoring an intervention to differing needs and circumstances.

While the findings of this study clearly indicate that the consequences of stroke influence stroke survivors’ sedentary behaviour, pre-stroke attitudes and routines can also play a significant role. This finding aligns with findings from a recent analysis of pooled data from nine studies (*n* = 274) that measured sedentary behaviour using the ActivPAL; only 11–19% of the variance in total sedentary time and time in prolonged sedentary bouts was accounted for by demographic and stroke-related variables [36].

### When and how to intervene to reduce sedentary behaviour after stroke?

Approximately half of the stroke survivors interviewed indicated that they would be interested in reducing their sedentary behaviour or making other lifestyle changes. The ‘teachable moment’ literature proposes that people are particularly open to making behavioural changes following major life transitions or health events, such as having a stroke [37]. Our findings suggest that views on the most appropriate time-point for intervention differ between stroke survivors, depending on their post-stroke recovery trajectory and related (perceived and actual) physical and psychological capability to reduce sedentary behaviour.

Providing stroke survivors with a rationale for reducing sedentary behaviour may enhance their motivation to increase standing and moving. Information about the health risks of sedentary behaviour– in particular the emerging evidence linking increased levels of sedentary behaviour to risk factors for recurrent stroke [38] – might also be motivating, as the fear of having another stroke is a common concern amongst stroke survivors [39]. Further, the findings of the present study suggest that stroke survivors are largely unaware of the potential health benefits of reducing sedentary behaviour. Nevertheless, the findings highlight that some, typically older, stroke survivors were not motivated by the potential health benefits of reducing sedentary behaviour, instead preferring to engage in activities they find enjoyable. To foster wider engagement, interventions to reduce sedentary behaviour after stroke should suggest engaging in, and provide examples of, meaningful and enjoyable activities involving standing and moving. Additionally, given that many stroke survivors value seated activities such as reading, interventions might specifically target reductions in ‘passive’ sitting (i.e. sitting whilst not engaging in any cognitive or social activity), and suggest taking breaks from enjoyable activities performed in a seated posture, rather than reducing the time spent engaged in these activities. Stroke survivors voiced a desire to return to independent and enjoyable activities that they engaged in prior to their stroke. Similarly, a recent qualitative study exploring physical activity in community-dwelling stroke survivors, found that participating in daily physical activity was viewed as beneficial for decreasing dependence on others and enhancing recovery from stroke-related motor impairments [40]. Thus, it may be pertinent to position standing and moving more as contributing to stroke recovery and increased independence.

Existing literature recognises that informal caregivers (i.e. family members) routinely support stroke survivors when they return home [41, 42] and that caregivers desire to be recognised as a stakeholder in the stroke survivors’ recovery [20]. Our findings indicate that caregiver support to complete activities can enable the stroke survivor to engage in activities they would not be safe or capable of performing alone. Caregivers’ narratives in the present study suggest that supporting and encouraging the stroke survivor to reduce sedentary behaviour could be an acceptable addition to their caregiver role, for those that do not have various other roles and responsibilities competing for their time. However, to do so, some caregivers would require support or advice about how and when to withdraw from doing things for the stroke survivor, and to move towards doing things with or supervising the stroke survivor and encouraging them to stand and move more. These findings align with findings from a qualitative study of physical activity after stroke, which reported that whilst caregivers can provide direct and indirect physical assistance to stroke survivors when needed, caregivers can also be overprotective, which is a barrier to stroke survivors moving [40]. Further, some stroke survivors do not have access to support from family and friends, and caregivers’ capacity to support an intervention can vary over time, and caregivers perceive that advice can sometimes be better received by stroke survivors when delivered by medical staff, so interventions should not solely rely on caregivers to support the stroke survivor.

### Strengths and limitations

This study adds to the limited existing literature exploring stroke survivor perspectives of (reducing) sedentary behaviour [19]. A strength of this study is the combination of interviews with observations, as observations help contextualise interview data and permit the collection of data within naturalistic settings [43]. Given that many stroke survivors require support or supervision to stand and move safely, establishing the views of stroke survivors’ informal caregivers to understand the barriers and facilitators to supporting an intervention to reduce sedentary behaviour was important. A noteworthy strength is the *theoretical* analysis of factors influencing stroke survivors’ sedentary behaviour. Utilisation of the COM-B model will aid in the development of empirically *and* theoretically informed behaviour change intervention. The COM-B model was used as this permitted the findings from this study to be fed directly into the behavioural analysis that comprises the first stage of intervention development following the BCW approach. Additionally, recruiting a heterogeneous sample of stroke survivors in relation to their social characteristics (age, gender, occupational status), time post stroke, and self-reported levels of sedentary behaviour permitted a broader range of perspectives to be included within the analysis. However, few participants had severe post-stroke impairments and all interviewees were ambulant, so the findings reported here are unlikely to represent the experiences of people who have had severe strokes.

Notwithstanding the observation component of the study, a limitation is that interviews were only conducted at 6-months or 9-months post-stroke; interviewees sometimes found it difficult to recall their experiences from the more immediate months following their stroke, i.e. when they were in hospital or in receipt of community stroke services, which is when a stroke-service intervention would be delivered. Stroke survivors and caregivers were interviewed together, which may have influenced their responses. All interview participants were community-dwelling, meaning that findings will not represent the experiences of those living in care homes or nursing homes. Whilst it was not an inclusion criteria for interview participants to be ambulatory, most of them were, meaning that the findings will not be generalizable to non-ambulatory stroke survivors. Member checks were not explicitly conducted with interview participants, however a lay summary of findings was shared with all participants and we did not receive any feedback to indicate that the findings did not resonate with their experiences. Additionally, as this study was undertaken in only two services the reported findings may not represent the experiences of stroke survivors and caregivers in other settings and countries.

## Conclusions

This qualitative interview and observation study examined factors influencing sedentary behaviour and propensity to reduce sedentary behaviour after stroke. The observational and interview data indicated that daily totals and patterns of sedentary behaviour varied between stroke survivors due to differing levels of capability, opportunity and motivation. Thus, interventions to reduce sedentary behaviour after stroke should be tailored to individual needs and circumstances or have the potential to be delivered flexibly. Key barriers identified by stroke survivors and their caregivers, and intervention ideas generated during this research, were fed into a co-production process involving stroke survivors, their caregivers, stroke service staff, and researchers, underpinned by the BCW, to develop an intervention to reduce sedentary behaviour in people after stroke. The intervention will be refined as part of a feasibility study (2019–2020) and evaluated in a 34-site cluster randomised controlled trial with an accompanying process evaluation (2020+).

## Supplementary information

**Additional file 1.**

**Additional file 2.**

**Additional file 3.**

## Data Availability

The dataset generated and analysed during the current study are not publicly available to reserve the anonymity of research participants.

## Abbreviations

BCW: Behaviour Change Wheel
COM-B: Capability, Opportunity, Motivation-Behaviour
FAC: Functional Ambulatory Categories
NEADL: Nottingham Extended Activities of Daily Living
SB-VAS: Sedentary Behaviour Visual Analogue Scale
