# Supplementary material for: Factors influencing sedentary behaviours after stroke: findings from qualitative observations and interviews with stroke survivors and their caregivers
Source: BMC Public Health. 2020 Jun 19;20:967. doi: 10.1186/s12889-020-09113-6 (PMC7305625; doi:10.1186/s12889-020-09113-6)
Supplement: Supplementary file 1 — Additional file 1. [file 12889_2020_9113_MOESM1_ESM.docx]

**Observational framework**

**Study title: Qualitative exploration of sedentary behaviours in stroke survivors using observation in stroke units and community services and interviews with patients, caregivers and staff (The RECREATE study)**

**Explanatory notes: non-participant observation framework**

This summary sheet refers to non-participant observations and documentary review only.

**General guidance relating to non-participant observations in stroke services:**

The purpose of this guidance is not to constrain data collection but rather to provide some common areas of initial and ongoing focus so that there is an agreed and overall structure for observational records. We anticipate that observational data collection will take place over 2 or more weeks in each site in each stroke service (an inpatient stroke unit and a linked community service). The researcher(s) will develop field notes and linked memos* which relate to but also extend and add to the areas of focus identified below and on the observation record sheet.

There will be two overlapping areas for non-participant observation, these are:

- General stroke service observations
- Patient, and staff member(s) observations (in stroke units and community stroke services)

**General stroke unit and community stroke service observations**

The purpose of these observations is to:

- develop understanding of the service, organisational and social context within which stroke care in general and more specifically sedentary behaviour related information, interaction and activity (sitting, lying, and anything to reduce or break up these) takes place
- develop understanding of the regularities and irregularities of the organisation, of work and of social interaction which enable understanding of how and why stroke patients may be sedentary (sitting, lying) across the day (either in the inpatient setting or in their own homes (part of the observations of community stroke services)
- identify the sedentary behaviour related activities, interactions, information provision or processes which are already occurring or opportunities where these could be introduced or enhanced

**Patient, caregiver and staff member(s) observations**

The purpose of these observations is to:

- identify, describe and explain the rehabilitation and sedentary behaviour related activities, events and interactions which represent therapist, rehabilitation assistant, other stroke team member, patient, family member or volunteer directed or supported activity in the selected stroke units or community stroke services;
- identify, describe and explain the contexts, facilitators, and barriers to providing and receiving sedentary behaviour related activity in the selected in-patient stroke units and community stroke services from the perspective of therapists, patients and stroke service managers;
- identify examples or indications of capability, opportunity and motivation to reduce/ break up sedentary behaviours;
- identify modifiable determinants of sedentary behaviour;
- identify and develop topics or questions to explore in semi-structured interviews with staff and former patients and their caregivers.

**Descriptions of rehabilitation-related activity categories:**

Physical activity: everyday, personal, recreational or occupational activities that require physical skills and utilize strength, power, endurance, flexibility, range of motion or agility. Examples include: sitting unsupported, standing, walking, actively transferring with or without assistance, upper limb (UL) exercises, dressing, eating and grooming.

ADLs (Activities of daily living): Every day activities generally involving functional mobility and personal care, such as bathing, brushing teeth, dressing, toileting and eating. (These activities are sometime referred to as PADLs – personal activities of daily living. For the purposes of this research we will use the term ADLs.)

DADLs (Domestic activities of daily living): These include household tasks such as shopping, cooking, laundry and cleaning.

Group activities: if observed in group activities, there is the potential for the patient to be engaged in a range of activity.

**Descriptions of sedentary behaviour related activity categories:**

Sedentary behaviours include: sitting, lying, reclining

Observe and record also: standing behaviours, and physical activity behaviours in the stroke units and linked community services;

Identify whether, how and in what context staff discuss the importance of reducing sedentary behaviour with stroke survivors and families in in-patient or community settings.

**Examples of good practice in field-note development** (Drawn from Spradley 1980 and with acknowledgement to Mary Godfrey at the Leeds Institute of Health Sciences)**:**

Spradley (1980) provides a useful format for thinking about and developing fieldnotes. He distinguishes between three different types of accounts.

1. A condensed account

It is very difficult to write down everything that everyone says and does as it happens. The notes made during observational fieldwork represent a condensed version of what actually occurred. These condensed accounts will often include phrases, questions, single words, numbers, and unconnected sentences and in some cases (e.g. meetings) diagrams indicating positions of people or the frequency of their contributions to the meeting. These are then expanded upon later.

1. Expanded account

Using the condensed account as an aide-memoire, the researcher should expand on the account adding details and what can be remembered. On each return visit to the field, the researcher need to observe with a view to examining recurrent events, noting similarities and differences (in personnel or in how they carry out the same activity for example) as well as the specific contexts in which different things happen. These provide important clues to the culture of the organisation/ phenomenon under investigation.

1. A fieldwork diary

In addition to fieldnotes that come directly from observing and informal interviewing (the condensed account and the expanded account); the researcher should also keep a reflexive diary or journal. This is the researcher’s own account of experiences, ideas, fears, mistakes, confusions, hunches, breakthroughs and problems encountered in the field. All diary entries should be dated. Commonly, experiences, impressions and questions merge into each other and it is easy to forget what was of paramount importance at the point an issue or question occurred to you. Later, in analysing and writing up the study, your fieldwork diary becomes an important source of data. It enables you to reflect on the process of collecting the data as a significant factor in what data was produced.

**Characteristics of the study site (record once for each Stroke service -update if significant change occurs during the observational period):**

| **Item** | **Specify/List** | **Comments** |
| --- | --- | --- |
| Urban or suburban location |  |  |
| Outline local catchment area population size and make-up |  |  |
| Identify and outline local and national policy initiatives or requirements impacting on the context and operation of the unit |  | E.g. Is there a hub and spoke model with centralisation of thrombolysis/thrombectomy services and repatriation to DGHs or is telemedicine used with thrombolysis in each DGH. How does this impact on length of stay?  What form does the community stroke service take (ESD like or alternative provision?). Is there in-reach or outreach community stroke provision? |
| Number of in patient beds or community caseload numbers |  |  |
| Average Length of Stay in inpatient settings (in days) or average number of days in receipt of community stroke service treatment |  |  |
| Staff Profile  List professional Groups (actual numbers as well as types),  Describe shift numbers (where appropriate), and/or management and organization of staff (including staff physical location) |  | Record here any information that will aid interpretation of the observational and interview data.  For example: is there 6 or 7 day working, what are the normal times of work for therapists, rehabilitation or therapy assistants and other members of the stroke unit or stroke service teams. |
| Record timing/frequency of routine activities:  For example therapy team meetings, MDT meetings,  Patient conferences, home visits, meal times, general ward rounds, work patterns across professions.  Record the equivalent processes for the community stroke service. |  | Therapy team or nursing staff meetings may be informal and occur at the beginning or end of each day with a summary of new patients or a review of the previous day’s activity and allocation of staff according to type of therapy required or number of staff required. |

| **Study title: RECREATE**  **Non participant observations: Common field-note record including guidance.** | | |
| --- | --- | --- |
|  |  | Comments (Use as for condensed fieldnotes and as the basis for development of expanded field-note records) |
| **Date** |  |  |
| **Unit Identifier** |  |  |
| **Researcher identifier** |  |  |
| **Visit number** |  |  |
| **Case identifier**  **(for case based observations only)** |  |  |
| **Total duration of non participant observation** |  |  |
| **Locations for observations (with time spent if appropriate)** |  | *Locations for general unit/service observations (stroke units and community stroke services) will include*  *-the therapy rooms or shared work station or any area where therapist and other stroke unit/community stroke service team members routinely congregate to discuss patient activity or meet with patients and carers*  *-staff meeting rooms (this can include attending multidisciplinary team meetings)*  *-For stroke units: therapy rooms/gymnasiums/occupational therapy kitchens or facilities which allow patients and carers to spend time independent of stroke unit staff in preparation for discharge home*  *-patient dining areas*  *-day rooms or other social communal areas*  *-bed areas*  *any additional areas where the researcher determines that it would be appropriate and gains consent to engage in non-participant observation*  *For community stroke services: the main focus will be on observing staff and patient and carer interactions and rehabilitation and sedentary behaviour (if evident) related activity in the participants’ own homes during routine community stroke service visits.*  ***Patient specific observations may also include most of the above areas, but will require verbal informed consent to undertake observations during specific therapy sessions or activities taking place in defined areas of the stroke units*** |
| **Focus of general non participant observation** |  | ***Areas of focus for general stroke unit observations will include:***  *-description of general activities routinely involving interaction between staff, patients and where appropriate with carers*  *-description of what appears to be important and meaningful for staff, patients in respect of rehabilitation or sedentary behaviour (if evident) related activity, for instance staff concerns, beliefs and preoccupations where these are verbalised*  *-description of specific activities focusing on independent practice or rehabilitation or sedentary behaviour (if evident) related activity with other members of the stroke unit team or patients and family members or volunteers*  *-description of the conditions under which patients, carers and staff members conduct their activities and interactions in the units, including perceived barriers and facilitators*  *-description of staff interactions*  *-description of informal unplanned activity, which appears to contribute to or reinforce rehabilitation or sedentary behaviour (if evident) related activity*  *-summary records of dialogue between participants may also be recorded when this is considered appropriate. Where verbatim recording of dialogue is considered important then written informed consent will be required from the participants. In the case of recurring dialogue, content which relates broadly to meeting the aims of the study, should not require consent.*  ***Additional areas of focus will clearly emerge in each study unit-researchers will develop field notes in these areas and should share/discuss these areas, with the other researchers generating data.*** |
| **Focus of patient-specific non-participant observation** |  | ***The patient based non-participant observations in stroke units and in participants’ own homes:*** *will include the above, (where appropriate) but here we are seeking more fine-grained and detailed description (and later explanation), which will aid in understanding:*  *Include-*  *-who was present*  *-the context of the rehabilitation or sedentary behaviour (if evident) related activity*  -*who leads and is participating in the rehabilitation* *or sedentary behaviour (if evident) related activity*  *-the nature and purpose of the rehabilitation or sedentary behaviour (if evident) related activity as articulated by the therapist, therapy or rehabilitation assistant, other member of the stroke team, patient, family member or volunteer.*  *-how the participants appear to respond to, participate in, feel about, describe, explain and make sense of the rehabilitation or sedentary behaviour (if evident) related activity*  *-the researcher's perceptions of the relationship of the activity to the aims of the study*  ***Please consider on each occasion:***  *Sedentary behaviours include: sitting, lying, reclining*  *Observe and record also: standing behaviours, and physical activity behaviours in the stroke units and linked community services;*  *Identify whether, how and in what context staff discuss the importance of reducing sedentary behaviour with stroke survivors and families in in-patient or community setting;.*  *Identify, describe and explain the contexts, facilitators, and barriers to providing and receiving sedentary behaviour related activity in the selected in-patient stroke units and community stroke services from the perspective of therapists, patients and stroke service managers;*  *Identify examples or indications of capability, opportunity and motivation to reduce/ break up sedentary behaviours;*  *Identify modifiable determinants of sedentary behaviour.*  ***Additional areas of focus will clearly emerge in each stroke service researchers will develop field notes in these areas and should share/discuss these areas, with the other researchers generating data.*** |
| **Documents reviewed in relation to observations only** |  | **Documentary review** *is designed to capture any textual information which will aid in understanding how rehabilitation and sedentary behaviour (if evident) related activity reported upon by stroke unit/community stroke service staff, patients, family members or volunteers.*  *Documents reviewed may include:*  *-information sheets or posters describing rehabilitation or sedentary behaviour (if evident) related activity or practice (directed individual activity) in the unit or information on involvement of patients and caregivers in rehabilitation or sedentary behaviour (if evident) related activity* |
| **Expanded field note record** |  | ***Detailed notes and reflections*** *of the researcher completed during the period of non-participant observations or documentary review (these will of course be written up following the period of observation).*  *Researchers may choose to record (anonymised) fieldnotes and reflections in notebooks or a password protected or encrypted digital recording device in the field, but overall observation records should be completed and saved (securely) electronically in NVivo using the headings identified in this document* |
| **Linked memo* number** |  | ***Researchers will be encouraged to record and develop memos***  *Memos are not simply "ideas." They are involved in the formulation and revision of explanations for the processes observed and later in the development of theory during the research process. Writing theoretical memos is an integral part of doing qualitative field research. See additional guidance on memo writing.* |

Blank template- routine recording of observations

| **Study title: RECREATE**  **Common field-note record** | | |
| --- | --- | --- |
|  |  | **Comments** |
| **Date** |  |  |
| **Unit Identifier** |  |  |
| **Researcher identifier** |  |  |
| **Visit number** |  |  |
| **Case identifier**  **(for case based observations only)** |  |  |
| **Total duration of non-participant observation** |  |  |
| **Locations for observations (with time spent if appropriate)** |  |  |
| **Focus of non-participant observation** |  |  |
| **Documents reviewed in relation to the patient based observations only** |  |  |
| **Expanded field note record** |  |  |
| **Linked memo number** |  |  |
| **Other relevant information** |  |  |
|  |  |  |

**Descriptive question matrix (after Spradley, 1980)**

| **Dimensions**  **(are interrelated)** | **What the observer might focus on** | **Example-** these provide the basis for more developed descriptions of individual patient treatments or activities which expand on the core areas identified below. |
| --- | --- | --- |
| SPACE | Description all of the spaces in which the phenomenon is occurring; what kinds of locations, physical structures and spatial ordering are evident? | The treatment area is approximately 15' x 25' and contains three plinths which can be easily moved. The arrangement of curtains allows sufficient privacy for up to three patients to be treated at the same time. This area can only be accessed via two sets of security locked doors from the main ward |
| OBJECT | Identification of the different objects, how are these utilised within the spaces which form part of the observations? | A large amount of equipment is stored down the left-hand side of the room; this includes wheelchairs, two freestanding mirrors, walking frames and sticks, plastic cones and wash bowls. These are selectively introduced by therapists, based on individual patient need during treatments. |
| ACT | Description related to what kinds of ‘acts’ are occurring on a regular, routine and infrequent basis. How are these linked to overall activity, and how are objects used, in particular spaces at different times by different actors? | A physiotherapist introduces a large exercise ball, placing the patient’s outstretched left arm over the top of the ball. An assistant repeatedly prompts the patient to keep his arm on top of the ball during the activity. |
| ACTIVITY | Identification of the range of activities occurring at different times, what kinds of activities are taking place and what is their goal or goals? | This patient has problems with sitting balance following his stroke. The physiotherapist and two assistants arrange themselves to sit one on either side and one behind the patient apparently to provide stability and support but also to assess the activity of particular muscle groups. |
| EVENT | Identification of single or multiple events, and whether they are related. How are different spaces are organised to facilitate events, do particular events occur at particular times are during particular time periods? | Attending to personal hygiene (washing and dressing) occurs in all of the ward areas and involves many different professional groups working singly or in pairs. The information overheard being given by team members often relates to sitting correctly. |
| TIME | Description which may include: what acts and activities occur at specific times and how these are related to the use of objects, in particular spaces? Closely related questions include whether different or the same actors are involved | Washing and dressing is the most common patient based event occurring between 0800 and 1000 everyday. During this time almost all members of the multidisciplinary team are involved in broadly the same activity. However, this takes place in a number of different spaces-the exception to this is medical staff who are present but engaged in visiting and assessing each patient. |
| ACTOR | Identification of individual actors, questioning and describing: where are they located in particular spaces, how do they use objects, and are their activities related to particular goals? | Nurses are almost exclusively found working in and around the bed spaces. In contrast, therapists and therapy assistance can be observed working in different spaces. These are determined in part by the kind of activity and the particular goal being worked on. Washing and dressing may occur at the bedside or in the therapy room and may involve a single therapist or two or more therapists working together. |
| GOAL | Description of the apparent purpose of events, and activities; do these change over time, is there a sequence to the activities? | Occupational therapists, describe washing and dressing as an important opportunity to determine patients functional abilities and to engage in support or exercises to improve upper limb function. Goals related to this activity are contributed to by physiotherapist and nurses, and sometimes involve relatives. |
| FEELING | Identification of different feeling states, which may occur in relation to different acts and activities. Do these differ between actors, events, and in terms of time and space? | Nurses and therapists talk of both frustration and satisfaction in relation to patients’ progress with rehabilitation. This is evident in individual treatment activities, where patients also express their feelings, and also in team meetings, where a range of feelings are expressed in relation to patient progression. |

**Observational fieldnote recording guidance**

| **Examples of observational fieldnote recording (after Patton, 2002)** | |
| --- | --- |
| ***Field Notes demonstrating insufficient detail*** | ***Field notes demonstrating more detailed description*** |
| 1. The physiotherapist and the occupational therapist helped the patient to wash their upper body. | The physiotherapist and occupational therapists brought the male patient to the therapy room in a wheelchair. He had experienced a stroke one week previously and had a marked weakness in his left arm and left leg. His speech was clear but he tended to look to the right most of the time unless except when asked to look at an object or person on his left. A low table had been prepared with personal washing and shaving items, a bowl and towels. The physiotherapist knelt on the floor in front of the patient, she took his left hand and then (saying ‘*look at me now Ron’*) explained that initially they were going to be working on improving his ability to sit up straight and support himself. They would also be placing a mirror in front of him so he could look to check on his balance. The physiotherapist said she would sit behind the patient and help him to get into the right position. The occupational therapist said once everyone was happy with his sitting position she would help him to get a wash and shave. |
| 2. The nurse gave out medicines to the patients taking to them as she did so. | A nurse is giving out medications; mostly these are from individual locked boxes on bedside lockers but some are in the trolley she is wheeling. She gently encourages a patient who seems confused to cover himself and repeats this request a number of times over the next 10 minutes. He allows her to cover him and he makes some unintelligible comments before picking at the bed clothes and gradually removing them again. She gives this patient some medicines via a tube feeding system but does not explain to him the actions she is performing. There is some conversation between the staff nurse and the other patients who are awake at this time; this precedes administration of individual medicines. Another nurse briefly enters the bay and goes directly to the confused patient, she gently and with apparent care, chastises him for removing the bed clothes, telling him she will come and wash and dress him first [before the other patients] because he *'can't keep pulling his clothes off like this'*. She leans over him with her face close to his and holds his hand whilst repeating her comments and puts her hand on his cheek and forehead. He responds with a smile to this touch and tries to verbalise in return although again this is unintelligible. She checks on one other patient and acknowledges each of the others... ‘*mornin’ Bob,* and *how are you Alan?* etc...' |

*The examples above provide an indication of the kind of descriptive observational recording, which should take place during fieldwork. These will be added to in theoretical memos and writing up and analysis of the observations made.*

**Condensed account with some pointers for analysis**

| Observations | Notes and analysis |
| --- | --- |
| I'm included in the ward round discussions for each patient although I feel a little out of my depth and with little to contribute at this stage. There seems to be a clear structure to reviewing each patient. The SHO and ward sister provide diagnoses, current medication, results of diagnostic tests. Functional and cognitive ability are measured by scales such as the Barthel, reports from therapists, observations of nurses and reports of family members are provided. I'm struck by a number of things:  -the range and complexity of the technical language, and how this is used by those present on the round (I begin writing lists of terms/test/scales I don’t understand).  -the discursive nature of the interaction, but with a sense of direction coming from the consultant physician if certain information is not forthcoming.  -the physical contact made with each patient (a handshake, a hand on the shoulder or holding a patients hand), social contact is established before progress is discussed  My inclusion in these discussions makes me feel more welcome but also points out my lack of understanding of the technical language shared by the team members. | 1) The process seems routine but is thorough and appears to ensure that details are not missed- a comprehensive picture of the patient is developed.  2) Team members seem to know what is expected of them and the process is quick, but thorough.  3) Clear attempts are made to engage the patients (there is conversation rather than simply declaring what will happen or what has been decided.).  The technical language is like a form of shorthand- how long does it takes to learn this and how important it is in feeling/being part of the team?  Roles seem to be understood, and I am struck by the discursive nature of the interaction between the team members on the round. This may be for my benefit, but it appears natural and was sustained for nearly 2.5 hours, also similar in the later team meeting |
