# Supplementary material for: Factors influencing sedentary behaviours after stroke: findings from qualitative observations and interviews with stroke survivors and their caregivers
Source: BMC Public Health. 2020 Jun 19;20:967. doi: 10.1186/s12889-020-09113-6 (PMC7305625; doi:10.1186/s12889-020-09113-6)
Supplement: Supplementary file 2 — Additional file 2. [file 12889_2020_9113_MOESM2_ESM.docx]

| Patient interview guide | **Interview questions** | **TDF** |
| --- | --- | --- |
| **Personal context** | | |
| 1. | Can you tell me a little about yourself and your home life?  (*Prompts: Who do you live with, employment status and previous employment, location of home, situation of home (flat / bungalow / house / supported living), paid and informal carers*) | n/a |
| 2. | Can you tell me a bit about when you had your stoke?  *(Prompts: How long since stroke, length of hospital stay, impact of stroke, whether this was 1^st^ stroke)* | n/a |
| 3. | How are you feeling generally?  (*Prompts: general health – how you feel, ability to do things around the home, ability to do things outside of the home, motivation to do daily tasks, motivation to do things for pleasure and enjoyment*) *any other recurring or current health issues)* | 4, 5, 10, 13 |
| 4. | What sorts of things are important to you in your recovery from your stroke?  *(Prompts: understanding your situation, getting well again, being able to go home or leave hospital, being able to move around again, regaining independence, spending time with family / friends / maintaining social networks, specific activities, e.g. going to bathroom unaided, preparing food, sport, driving, getting back to work?)* | 3, 6, 8, 9 |
| **Behavioural context – current behaviour, and the capability, opportunity and motivation of stroke survivors to reduce / break up sedentary behaviour** | | |
| - Check participant is happy with questions so far / OK to continue - Explain focus of next set of questions (sitting and lying activities they engage in) | | |
| 5. | Sitting and lying behaviours - could you describe a typical day?  *Note: adapt prompts based on their capability to mobilise e.g. ask about times sat out compared to lying, any upper body movement, changing positions.*   1. A. What do you do when you are sitting or lying down?   *(Prompts: hobbies, chatting, watching TV, reading, nothing, alone / with others, using a computer / tablet device, knitting, needlework or other craft based activity, work, travel)*  *B. Are these activities important in your daily life? Why are they important / not important?*  *(Prompts: enjoyment, linked to fulfilment of ‘roles’ including work-related, lifestyle / social, linked to values)*   1. A. What do you do that involves standing or movement?   *(prompts: domains e.g. hobbies or leisure (sports clubs, walking, shopping)/ household tasks (cleaning, cooking, dressing)/ travel (walking, bus)/work, who do you do these activities with, how often do you do these activities, do you require anything specific to do it (materials, equipment, another person)*  B. Are these activities important in your daily life? Why are they important /  not important?  *(Prompts: enjoyment, linked to fulfilment of ‘roles’ including work-related, lifestyle / social, linked to values)*   1. Are there times of the day you sit or lie down more or less? Are there days when you sit or lie down more than others?   *(Prompts: weekdays vs. weekend days, sitting in different contexts including work, travel, leisure and household – where does most / least sitting occur?)*   1. Do you monitor how much time you spend sitting and lying down?   (*Prompts: Are you aware of how much time you spend sitting, (if yes) how do you monitor this, do you ever feel like you’ve been sitting for too long? Why do you feel this?, do you plan ahead about when you might need to get up to sit / lie down more (certain times of the day or after a certain amount of time has passed / to do certain activities)*   1. *Would you say the amount of time you spend sitting and lying down has changed over the years?*   *(Prompts: how has it changed (more / less / pattern), change in types of behaviour, due to what factors such as health / retirement, how do you feel about it)* | 2, 3, 8, 10, 14 |
| 6. | Now I am going to ask some questions that focus on your views on the benefits and drawbacks of sitting and lying down   1. Have you noticed a difference in the way you feel when you have been sitting for a lot of the day compared with when you have been up and moving about?   *(Prompts: in what way (mentally, physically), why do you think this is?)*   1. What do you think are the benefits of sitting or lying for a long time?   *(Prompts: feeling safe, not pushing yourself / ‘rest is best’, being able to do activities you enjoy most, reduced fatigue / comfort when sat)*   1. What do you think are the drawbacks of sitting or lying for a long time?   (*Prompts: how does prolonged sitting / lying affect your health (mental / physical / emotional), reduced capability to stand/walk if don’t practice and relationship to falls / risk of falls, boredom)*   1. What do you think are the benefits of reducing or breaking up sitting and lying behaviours?   (*Prompts: linked to rehabilitation and meaningful activities, being able to do things such as housework, leaving the house, socialising)* | 1, 5, 6, 13 |
| - Check participant is happy with questions so far / OK to continue - Explain focus of next set of questions (factors that make breaking up sitting and ling behaviours / movement easier or harder) | | |
| 7. | Have you made any changes to your lifestyle since you had your stroke?  *(Prompts: beneficial changes - what decisions (e.g. stopping smoking, reducing alcohol intake, improving diet, taking up exercise), what specifically prompted these decisions, did you act on the decision / sustain the lifestyle change (if so, how long did it take to form the habit?), did you set any goals, has it been the correct decision, what benefits have you noted since making these changes; sit more – did stay in hospital contribute to this? In what way?, where did you get the information to support you to do this?)* | 1, 2, 6, 7, 8, 9, 10, 12, 14 |
| 8. | Could you sit less / lie down less throughout the day? Would this be difficult?   1. How do you find: getting out of bed, getting out of a chair, going outside?   (*Prompts: any difficulties (e.g. physical ability, motivation to do so, local environment – barriers in the home / outside of the home, need for assistance from another person)*   1. Do you think your stroke has influenced the amount of time you spend sitting? Has it influenced how easy it would be to sit less throughout the day?   *(Prompts: causes, symptoms, consequences – vision, hearing, cognitive issues such as attention and understanding, mood/motivation, physical ability to use upper and lower limbs, fatigue, pain, anxiety/fear, other (non-stroke related) health issues)*   1. If a health professional suggested that you would benefit from reducing or breaking up the time you spend sitting or lying, how would that make you feel?   *(Prompts: e.g. anxious, annoyed, motivated, concerned, confused, hopeful, interested, angry )* | 2, 4, 10, 12, 13, 14 |
| 9. | Are there any other factors that influence how long you sit or lie down?   1. Let’s start with things in your home environment   *(Prompts: aspects that influence how much you currently sit – stairs / whether live alone / location (rural / urban), how it might be changed to encourage reduced sitting)*   1. Thinking now about outside of your home: What about the influence of your local area?   *(Prompts: can you drive or is there anyone to drive you, are you well connected to bus routes, neighbourhood walkability (provision of paths, road crossings, perception of safety), are there adequate spaces / places / activities within your community, cost of travel and activities)*   1. How about the influence of other people?   *(Prompts: influence they have on current behaviour e.g. is sitting with friends and family a regular part of your day / do they encourage you to sit / rest and do things for you, role they could play in supporting sitting reduction, what they might think about you sitting less, thinking about people including family and friends / paid carers / NHS staff and volunteers, how did this input make you feel?)*   1. What services have you been given information about / attended following your stroke? Has use of services (including the stroke service) influenced how much you sit or lie down?   *(Prompts: services including stroke service / befriending service / community groups, do/did they directly encourage or contribute to reduced sitting, did/have they offered any advice to sit less or that would conflict with advice to sit less do you travel to them or do they necessitate you staying indoors)* | 11, 12 |
| **Future intervention** | | |
| - Check participant is happy with questions so far / OK to continue - Explain focus of final questions (thoughts on how to sit and lie down less) | | |
| 10. | Would you like to spend less time sitting or lying down?  (*Prompts: how much less time, how important is this to you, do you know how to go about this*) | 8, 9, 13, 14 |
| 11. | What sorts of things, realistically, do you think you could do to sit and lie down less?  *(Prompts: could you incorporate more standing / movement into the activities you currently do and enjoy (discussed earlier), how would you go about doing this, prompt to think about reducing and breaking up sitting rather than increasing PA, e.g. standing when on the phone)*   1. What prompts could you use to help you?   *(Prompts: standing during TV adverts, comfort, time-based, alarms, social interactions, wall planner)*   1. Are there any challenges that might prevent you from doing these things? 2. How might these be overcome? | 1, 7, 10, 11, 14 |
| 12. | 1. What would help you to do less sitting?   (*Prompts: through and with which other people e.g. frien11, 12ds / NHS staff / stroke survivors, being active, group classes (stroke specific or not), individual interventions, advice on discharge, follow up from stroke outreach services*)   1. When would it be best to encourage people to do less sitting?   *(Prompts: in-patient / community stroke service / alternative route, time since stroke, stage of rehabilitation, time of day )*   1. Would you be interested in taking part in a programme that supported you to sit/lie down less? Why / why not? | 11, 12 |
| 13. | Is there anything else that you’d like to add / that you feel we’ve not discussed? | 4, 8, 11 |

TDF domains key – patient interview guide

1. Knowledge –3 questions
2. Skills – 2 questions
3. Role and identity – 2 questions
4. Beliefs about capabilities – 3 questions
5. Optimism – 2 questions
6. Beliefs about consequences – 3 questions
7. Reinforcement – 2 questions
8. Intentions – 5 questions
9. Goals – 3 questions
10. Memory and decision processes – 5 questions
11. Environmental context – 4 questions
12. Social influences – 4 questions
13. Emotion – 4 questions
14. Behavioural regulation – 5 questions

Caregiver interview guide

|  | **Inteview question** | | **TDF** |
| --- | --- | --- | --- |
| **Caregiver context – responsibilities as a caregiver and interactions with stroke survivor** | | | |
| 1. | Who do you provide care for?  *(Prompts: relationship to patient, whether primary / sole caregiver and who else is involved in providing care to patient, amount of time per day / week spent acting as a caregiver (e.g. full / part / days per week)), whether live with patient / location)* | n/a | |
| 2. | Can you tell me a little about the caring activities you undertake?  *(Prompts: What being a caregiver entails or ‘day in the life’, patient contact (typical interaction – length, purpose, dialogue, place, others present, equipment required, challenges), contact with stroke service staff, contact with other caregivers, how different support organisations / staff work together, any specific or special requirements of being a caregiver)* | n/a | |
| 3.. | How long have you been providing care (to stroke survivor)?  *(Prompts: prior to / since the event of the stroke, other roles and responsibilities (e.g. caring for other adults / children, employment, own health), any specific learning / (life) changes that have been required in order to act as a caregiver)* | n/a | |
| 4. | What sort of things do you perceive to be important to the stroke survivor you care for, in relation to their recovery from stroke?  *(Prompts: understanding their situation, getting well again, being able to move around again with reduced / limited / no assistance, regaining independence, spending time with family / friends, specific activities (e.g. going to bathroom unaided, preparing food, sporting activities?), returning to work)* | 3, 6, 8, 9 | |
| **Behavioural context – current practice / behaviour, and the capability, opportunity and motivation of caregivers to encourage stroke patients to reduce / break up sedentary behaviour** | | | |
| - Check participant is happy with questions so far / OK to continue - Explain focus of next set of questions (sitting and lying behaviours of yourself and stroke survivor you care for) | | | |
| 5. | (a) What sorts of activities do you do during the day?  (*Prompts: types of activities you do (e.g. ADL, leisure work, travel, any physical activities/exercise/sport), whether sat/lying down doing these, frequency, who you do these activities with, importance of these activities (sitting and non-sitting), whether sitting time/patterns have changed over years and since relative had stroke, how do you feel about sitting less)*  (b) What role do you have in encouraging the stroke survivor you care for to reduce or break up the time they spend sitting and lying?   1. Do you do anything that enables / supports the stroke survivor you care for to reduce or break up the time they spend sitting or lying?   *(Prompts: supervising / physically supporting movement, taking / supervising stroke survivor on activities (e.g. leaving home, hobbies, housework, therapy)*   1. What is the main aim of these activities?   *(Prompts: is the main aim to break up sitting or something else e.g. therapy, hobby, ADL)*   1. How often do you do these things?   *(Prompts: frequency of such behaviour/is this something you routinely do, sporadic / regular basis, established routines, recommended activities, formal / informal)*   1. Do you say things to the stroke survivor you care for to encourage them to reduce or break up the time they spend sitting or lying?   *(Prompts: What do you say? How do you motivate them? E.g. incentives, linking to recovery, benefits of breaking up sitting?)* | | 2, 3, 8, 10, 14 |
| 6. | Now I’m going to ask some questions that focus on your views on the benefits and drawbacks of sitting and lying down after having a stroke   1. What do you think are the benefits of sitting or lying for a long time after a stroke/for the stroke survivor you care for?   *(Prompts: feeling safe e.g. risk of falls or another stroke when not sat or lying, not pushing yourself / ‘rest is best’ for recovery, being able to do activities you enjoy most, reduced fatigue / comfort when sat)*   1. What do you think are the drawbacks of sitting or lying for a long time after a stroke?   *(Prompts: how does prolonged sitting / lying affect health (mental / physical / emotional) and wellbeing and recovery from stroke, reduced capability to stand/walk if don’t practice and relationship to falls / risk of falls, boredom)*   1. What do you think are the benefits of reducing or breaking up sitting and lying behaviours after a stroke?   *(Prompts: improved health and wellbeing, linked to rehabilitation and meaningful activities, being able to do things such as housework, leaving the house, socialising)*   1. Is encouraging the person you care for to spend less time sitting / lying a priority for you?   *(Prompts: within your caring ‘role’ and within your wider life and responsibilities: why/why not, what other tasks take priority, where does it rank in comparison to other tasks, is there anything that might be useful in encouraging reducing time spent sitting and lying to become a higher priority task)* | | 1, 5, 6, 13 |
| 7. | Are you aware of any lifestyle factors that can have an influence on health and wellbeing generally?  (*Prompts: e.g. – smoking, alcohol consumption, physical activity/sedentary behaviour, food choices)*   1. Have you encouraged the stroke survivor you care for to make any changes to their lifestyle since having their stroke? *(Prompts: e.g. – smoking, alcohol consumption, physical activity, food choices – IF YES - why, when, in what context; IF NO – why not? Would you feel comfortable/prepared to discuss issues with SS))* 2. If yes: How do you think this information was received? (*Prompts: positive / negative, acted upon / not acted upon, misunderstood, needed some persuasion, keen to implement, does the SS you care for value your input/advice when making decisions more generally)* | | 1, 6, 7 |
| - Check participant is happy with questions so far / OK to continue - Explain focus of next set of questions (factors that make breaking up sitting and ling behaviours / movement easier or harder) | | | |
| 8. | Could you encourage the stroke survivor you care for to sit / lie down less?   1. Do you feel you are able to support the stroke survivor you care for to reduce / break up time spent sitting and lying?   *(Prompts: knowledge / skill, your own physical health / strength /fatigue (to physically support), confidence, emotional capacity, consequences of sitting/lying less, environmental factors, time, other priorities, own approaches to sitting / lying)*   1. Can you identify any factors related to the stroke survivor that might influence how feasible it is for you to encourage them to reduce/break up time spent sitting and lying?   *(Prompts: physical ability to use upper and lower limbs, vision, hearing, cognitive issues such as attention and understanding, severity of stroke, co-morbidities i.e. other (non-stroke related) health issues, motivation, pre-stroke behaviour, mood/motivation, fear/anxiety, fatigue, pain, falls risk)*   1. How do these factors make you feel about encouraging the stroke survivor you care for to reduce or break up the time they spend sitting or lying down?   *(Prompts: Less or more willing/motivated, anxious/nervous/concerned – don’t want set-backs, happy, confident, satisfied with self / stroke survivor; have feelings changed over time – how did you feel initially following stroke / commencement of caring activities compared to how feel now; do your feelings differ with regards to encouraging SSs to break up sitting in the home environment vs. away from home)* | | 1, 2, 3, 4, 6, 10, 13 |
| 9. | Are there any other factors that influence how easy it would be for you to encourage the stroke survivor you care for to sit / lie down less?   1. Let’s start with things related to your own circumstances   *(Prompts: do you live with the stroke survivor – if not, how far away etc., do you drive, other responsibilities (e.g. work, children), sharing the stroke survivor’s care with others, extent of caring role (light touch vs full time) and how often see/speak to the stroke survivor, own views/attitudes about time spent sitting, time you spend sitting yourself, finances)*   1. Thinking about the home environment of the stroke survivor   *(Prompts: aspects that influence how much the stroke survivor currently sits – stairs / whether live alone / location (rural / urban) / things they do during the day, how it might be changed to encourage reduced sitting)*   1. Thinking now about outside the home   *(Prompts: are you well connected to bus routes, neighbourhood walkability (provision of paths, road crossings, perception of safety), are there adequate spaces / places / activities within your community, cost of travel and activities, how SS is supported outside of the home (e.g. do they need assistance to leave the house))*   1. How about the influence of other people?   *(Prompts: e.g. other friends/relatives of stroke survivor (do they contradict your beliefs/behaviours), advice given to you by your own friends/relatives, stroke service staff, paid carers, volunteers, neighbours, exercise class providers / instructors)*   1. How about the availability and quality of services?   *(Prompts: services including stroke service / befriending service / community groups, do/did they directly encourage or contribute to reduced sitting, online resources, exercise classes, did/have they offered any advice to sit less or resources to support carers to encourage SS to break up sitting, if not would this help you)* | | 3, 11, 12 |
| **Future intervention** | | | |
| - Check participant is happy with questions so far / OK to continue - Explain focus of final questions (thoughts on how to encourage stroke survivor you care for to sit and lie down less) | | | |
| 10. | What do you think would be possible for you to do to encourage stroke patients to reduce or break up time spent sitting or lying?  (*Prompts: integrate into usual activities, discuss with stroke patients, how would you prioritise these, utilising prompts, e.g. standing during TV adverts / comfort / time-based)*   1. What might be the challenges doing these things? 2. How might they be overcome? | | 2, 4, 5 |
| 11. | 1. What would help you to encourage the stroke survivor you care for to sit / lie down less?   (*Prompts: information / guidance about how to do this, knowing more about benefits/risks of breaking up sitting, support from which other people and services, group classes, objects/environmental adaptions/tools such as online resources or support networks, advice on discharge, follow-up from stroke outreach services*)   1. When do you feel it would be best to encourage the stroke survivor you care for to spend less time sitting and lying?   *(Prompts: in-patient / community stroke service / alternative route, time since stroke, stage of rehabilitation, time of day )*   1. Would you be interested in taking part in a programme to encourage the stroke survivor you care for to sit/lie down less? Why/why not? | | 11, 12 |
| 12. | Is there anything else that you’d like to add / that you feel we’ve not discussed? | |  |

TDF domains key – caregiver interview guide

1. Knowledge – 3 questions
2. Skills – 3 questions
3. Role and identity – 4 questions
4. Beliefs about capabilities – 2 questions
5. Optimism – 2 questions
6. Beliefs about consequences – 4 questions
7. Reinforcement – 1 question
8. Intentions – 2 questions
9. Goals – 1 question
10. Memory and decision processes – 10 questions
11. Environmental context – 2 questions
12. Social influences – 2 questions
13. Emotion – 2 questions
14. Behavioural regulation – 1 question
