# Supplementary material for: Factors influencing sedentary behaviours after stroke: findings from qualitative observations and interviews with stroke survivors and their caregivers
Source: BMC Public Health. 2020 Jun 19;20:967. doi: 10.1186/s12889-020-09113-6 (PMC7305625; doi:10.1186/s12889-020-09113-6)
Supplement: Supplementary file 3 — Additional file 3. [file 12889_2020_9113_MOESM3_ESM.docx]

**Capability**

Stroke survivor: 3.3 (standing and movement capability after stroke)

The COM-B model, including an illustration of how the findings align with the model

**Opportunity**

Stroke survivor: 3.2 (the physical and social environment in the stroke service and in the home)

Caregiver: 3.5.2 (the caregiver ‘role’, responsibilities and circumstances)

**Motivation**

Stroke survivor: 3.4 (emotions and motivation after stroke), 3.6.1 (intervening to reduce sedentary behaviour after stroke)

Caregiver: 3.5.3 (perceived consequences of supporting sedentary behaviour reduction)

**Behaviour**

Stroke survivor: 3.1 (sedentary behaviour levels and patterns after stroke)

Caregiver: 3.5.1 (current caregiver support behaviour)

Stroke survivor – theme 3.3

SS – themes 3.4, 3.6.1

C – theme 3.5.3

SS – theme 3.2

C – theme 3.5.2

SS – theme 3.1

C – theme 3.5.1
